# Interference in Pheromone-Responsive Conjugation of a High-Level Bacitracin Resistant *Enterococcus faecalis* Plasmid of Poultry Origin

**DOI:** 10.3390/ijerph10094245

**Published:** 2013-09-11

**Authors:** Cindy-Love Tremblay, Marie Archambault

**Affiliations:** GREMIP research group, Department of Pathology and Microbiology, Faculty of Veterinary Medicine, University of Montreal, 3200 Sicotte Street, Saint-Hyacinthe, QC J2S 7C6, Canada; E-Mail: cl.tremblay@umontreal.ca

**Keywords:** *Enterococcus faecalis*, pheromone-responsive conjugation, plasmid, bacitracin resistance genes, antiserum AS

## Abstract

The current study reports on contact interference of a high-level bacitracin- resistant pheromone-responsive plasmid of *Enterococcus faecalis* strain 543 of poultry origin during conjugative transfer of *bcr* antimicrobial resistance genes using a polyclonal antiserum aggregation substance_44–560_ (AS). After induction with pheromones produced by the recipient strain *E. faecalis* JH2-2, clumping of the donor *E. faecalis* strain 543 was observed as well as high transfer frequencies of *bcr* in short time broth mating. Filter mating assays from donor strain *E. faecalis* 543 to the recipient strain *E. faecalis* JH2-2 revealed conjugative transfer of *asa1* (AS), *bcrRAB* and *traB* (negative regulator pheromone response) genes. The presence of these genes in transconjugants was confirmed by antimicrobial susceptibility testing, PCR, Southern hybridization and sequencing. A significant reduction in formation of aggregates was observed when the polyclonal anti-AS_44–560_ was added in the pheromone-responsive conjugation experiments as compared to the induced state. Moreover, interference of anti-AS_44–560_ antibodies in pheromone-responsive conjugation was demonstrated by a reduction in horizontal transfer of *asa1* and *bcr* genes between *E. faecalis* strain 543 and *E. faecalis* JH2-2. Reducing the pheromone-responsive conjugation of *E. faecalis* is of interest because of its clinical importance in the horizontal transfer of antimicrobial resistance.

## 1. Introduction

*Enterococcus faecalis* is part of the normal animal and human gut flora. It is also a major cause of nosocomial infections in humans [1] and has been linked to severe extra-intestinal infections in poultry [2]. Clinical conditions observed in poultry include pulmonary hypertension syndrome [3], and amyloid arthropathy [4], in addition to first week mortality [2]. Also, a study has demonstrated the high potential of a few *E. faecalis*-contaminated eggs or embryos to rapidly facilitate the spread of this bacterium to almost all chickens during hatch [5]. More recently, it was demonstrated that *E. faecalis* of human and poultry origin shared virulence genes supporting the zoonotic potential of *E. faecalis* [6]. 

*E. faecalis* is able to acquire antimicrobial resistance through transfer of plasmids and transposons, chromosomal exchange, or mutation and this presents a significant challenge for therapeutic measures [7]. Poultry *E. faecalis* was identified as a possible antimicrobial resistance reservoir for human communities for glycopeptides (vancomycin and avoparcin) antimicrobials in European countries [8]. In some cases, the vancomycin resistant enterococci (VRE) were shown to persist in slaughtered poultry many years after the ban on avoparcin [9]. Enterococci transferable genetic elements have a broad host range and can even be transferred to both gram-negative and gram-positive bacteria [10]. Thus, *E. faecalis* could also potentially act as a source of antimicrobial resistance genes for important poultry intestinal pathogens. Conjugation systems involving plasmids and transposons are abundant in enterococci [10]. The sex pheromone response system [11], which is highly specific to *E. faecalis*, has been associated with virulence and antimicrobial resistance [10,12]. This system involves the production of sex pheromones by the recipient and the subsequent recognition of these pheromones by the donor. Each pheromone is specific for a particular plasmid or group of related plasmids [13]. Two major cell surface proteins are synthesized after induction with the appropriate sex pheromone, an adhesin or aggregation substance (AS), that enables contact between donor and recipient during conjugation, and the surface exclusion protein which reduces plasmid transfer between the donor cells harbouring the same sex pheromone plasmid [14]. The AS binds to a receptor on the surface of the recipient cell to form aggregates and to enhance the conjugation process [15].

An ABC transporter system involved in bacitracin resistance, in addition to an overproduced undecaprenol kinase were described in *E. faecalis* [16]. These two mechanisms are both plasmid (pJM01 of ca. 72 kb) encoded by the *bcrABD* operon, which in turn is under the control of a regulatory gene, *bcrR*. It was suggested that this plasmid was pheromone-responsive since negative regulator protein associated genes of the pheromone response, *traA* and *traB,* were detected [17]. Another pheromone-responsive plasmid of ca. 85 kb, named pTW9, encoding for bacitracin (*bcrABD* operon), vancomycin (*vanA* operon) and MLS resistance genes (*erm*(B) and *erm*(C)) was recently described in *E. faecalis* (accession number AB563188).

Little information is available on contact interference between donor and recipient cells during pheromone-responsive conjugative transfer of plasmids encoding antimicrobial resistance genes. Interference in the pheromone-responsive conjugation was first described by Olmsted *et al.* [15] using specific F’ab fragments to the AS, named Asc10, of the pheromone-responsive plasmid pCF10. The presence of F’ab fragments reduced the mating frequency by a factor of about 100 which was also correlated with the inhibition of aggregation [15]. In addition, various transfer levels were observed for different insertion Asc10 mutants, and transfer levels correlated with aggregation ability [18]. Reducing the pheromone-responsive conjugation of *E. faecalis* is of interest because of its clinical importance in the horizontal transfer of antimicrobial resistance.

The purpose of this study was to report on contact interference of a high-level bacitracin resistant pheromone-responsive plasmid related to pJM01 in an *E. faecalis* strain of poultry origin during conjugative transfer of its *bcr* antimicrobial resistance genes using a polyclonal antiserum AS.

## 2. Material and Methods

### 2.1. Bacteria, Media and Antibiotics

The previously described *E. faecalis* strain 543 [19] and *E. faecalis* JH2-2 [20,21] were used in this study. Briefly, *E. faecalis* strain 543 is positive for the AS gene, *asa1*, resistant to bacitracin, ciprofloxacin, erythromycin, gentamicin, kanamycin, nitrofurantoin, streptomycin, tetracycline, and tylosin with the resistance genes *bcrR, bcrA, bcrB, aadE, tet*(M), *tet*(O), and *erm*(B). *E. faecalis* JH2-2 is a fusidic acid- and rifampin-resistant strain and a well characterized pheromone-producer [20,21]. Brain heart infusion broth (BHI) (Oxoid, Thermo Scientific, Nepean, ON, Canada), trypticase soy broth and agar (TSB and TSA) (Difco, Fisher Scientific, Ottawa, ON, Canada) and blood agar (TSA plus 5% sheep blood) were used for routine growth. Isolates were maintained in glycerol at −20 °C before testing. For the donor and the transconjugants, antibiotics were used at the following concentrations in TSA: fusidic acid, 25 µg/mL; rifampicin, 25 µg/mL; bacitracin, 100 µg/mL; and tetracycline, 10 µg/mL (Sigma-Aldrich, Oakville, ON, Canada).

### 2.2. PCR, Specific Primers and DNA Sequencing

Detection of the AS gene *asa1* and the bacitracin resistance genes (*bcrR, bcrA* and *bcrB*) was performed by PCR with primers and conditions previously described [16,22] with minor modifications. Briefly, to 5 µL of DNA was added 2.5 µL of 10× PCR Buffer, 0.2 mM of dNTPs, 2 mM of MgCl_2_, 200 mM of each primers and 1.25 U of *Taq* DNA polymerase (GE Healthcare, Québec city, QC, Canada) in a total volume of 25 µL. New primer constructions (5′ to 3′) were as follows: *bcrR* forward tatagggttctcttgccgct, *bcrR* reverse gttaccctaacatggagtcg, *bcrA* forward aatccgtcatgttggtagctgctct, and *bcrA* reverse tattatgcacgagccggagcttct. Detection and sequencing of the pheromone shutdown protein associated-gene *traB*, and regions above *bcrB* and beyond *bcrR* were performed by PCR with the following primers (5′ to 3′): *traB* forward gtgctgaagacgtgggggctg, *traB* reverse accggcgacagtgcacctact, above *bcrB* forward gccgtttcatgggcgtgaaa, above *bcrB* reverse acctttcaccatttcaaaaaggagg, beyond *bcrR* forward tcctgcgttaagttctttccagtcc, beyond *bcrR* reverse tcctgcacttcacgataactcaggt. DNA sequencing was performed on an ABI PRISM 310 Genetic Analyzer (Applied Biosystems, Concord, ON, Canada). Homology searches using BLAST were performed through the NCBI website [23].

### 2.3. Filter Mating

Filter mating experiments were performed as previously described [24] with some modifications. *E. faecalis* JH2-2 was used as a recipient strain in mating experiments with the donor *E. faecalis* strain 543. Donor and recipient cultures were mixed in a 1:1 ratio and added on a 0.22 µm-pore-size filter (Fisher Scientific) then placed on a blood agar plate and incubated for 24 h at 37 °C. Transconjugants were selected on TSA agar containing the appropriate antimicrobials.

### 2.4. Pheromone Induction and Aggregation

The detection of aggregation (clumping) was performed as previously described [10] with minor modifications. An overnight culture filtrate of plasmid-free *E. faecalis* JH2-2 was used as the pheromone. Briefly, clumping was evaluated by adding 100 µL of overnight-cultured *E. faecalis* strain 543 to 0.9 mL of pheromone previously diluted (1:2) in fresh BHI broth. The mixtures were incubated at 37 °C for 90 min with shaking, mounted on glass slides, and observed by microscopy for clumping (Leica DMI4000 B Inverted Microscope, Meyer Instruments, Houston, TX, USA). Clumping was recorded as the numbers of aggregates per field. Negative controls were prepared by replacing pheromone-containing filtrates with BHI broth.

### 2.5. Pheromone-Responsive Conjugation Experiments

Short mating induced by the pheromone was performed as previously described [11] with minor modifications. After induction, 0.1 mL of the donor *E. faecalis* strain 543 was mixed with 0.1 mL of the recipient strain *E. faecalis* JH2-2 and the mixture was incubated for 20 min at 37 °C. Dilutions of the mixture were then plated on selective plates containing the appropriate antimicrobials and incubated 48 h. Transfer rates were calculated as transconjugants/donor ratio. Gene transfer was confirmed by PCR on transconjugants. To evaluate mobile genetic element stability without selective pressure, broth passages in TSB were performed as previously described [16] and genes lost was confirmed by PCR. One transconjugant (T543-1) was selected for further analysis.

### 2.6. Minimum Inhibitory Concentration (MIC) Determination

Antimicrobial susceptibility testing was performed using broth macro-dilution as described by the recommended Clinical and Laboratory Standards Institute (CLSI, M31-A3) guidelines on the donor strain *E. faecalis* 543 and the transconjugant T543-1 to determine their MIC to bacitracin [25].

### 2.7. Production of Antiserum and Interference in Pheromone-Responsive Conjugation

The *E. faecalis* AS_44–560_ protein expression, purification and production of antiserum were performed by GenScript Corporation (Piscataway, NJ, USA) according to their Basic Polyclonal Antibody Package (Rabbit). Sodium dodecyl sulphate-polyacrilamide gel electrophoresis (SDS-PAGE) and Western blot with the purified protein AS_44–560_ were performed to demonstrate specificity of the polyclonal anti-AS_44–560_ (GenScript Corporation). Efficacy of the third and fourth immunizations was determined with an enzyme-linked immunosorbent assay (ELISA) (GenScript Corporation). Interference in the conjugation process was performed with the addition of the polyclonal anti-AS_44–560_ (non-diluted, 1:5, 1:10) to 1 mL of phosphate-buffered saline 1× (PBS) containing donor cells and incubated for 90 min at 37 °C. Cells were washed four times with PBS 1× at 4 °C to remove non-specific binding and then resuspended in 0.9% saline for mating incubation (20 min, one and two hours). Gene transfer and transfer rates were evaluated as described above. Pre-immune serum (1:10) (GenScript Corporation) and non-induced bacteria were used as negative controls. Pheromone-induced bacteria incubated with PBS 1× without antibodies were used as a positive control. Experiments were performed in triplicates.

### 2.8. DNA Microarrays

Microarrays were performed on donor strain 543, recipient JH2-2 and one of their transconjugates as previously described by Champagne *et al.* [26]. Labelled DNA was hybridized on an antimicrobial resistance microarray, containing 173 antimicrobial resistance genes, 15 virulence factors and 70 taxonomic probes, developed for *Enterococcus* [27] at the Biotechnology Research Institute in Montreal (BRIM), Canada. The microarray contains also two added oligonucleotides encoding for bacitracin resistance (*bcrA* and *bcrB* genes), respectively. Arrays were analysed using a ScanArray microarray Scanner (model Express, Perkin-Elmer, Fremont, CA, USA) and the Scanarray Express software program version 1.1. Biological and technical replicates were included in the validation process. Oligonucleotides with a signal-to-noise fluorescence ratio above 3 were considered positive.

### 2.9. Pulsed-Field Gel Electrophoresis (PFGE)

The DNA plugs preparation and PFGE were performed as previously described [28,29]. Briefly, a plug slice of 2 to 4 mm wide was suspended in a total volume of 227 µL of the manufacturer’s recommended restriction buffer and 20 U of SmaI (New England Biolabs Inc., Mississauga, ON, Canada). Digestion mixture was incubated at 25 °C for 2 h. The gel was electrophoresed with a CHEF-DRII apparatus (Bio-Rad, Mississauga, ON, Canada) for 21 h at 5.8 V/cm with switching times ramped from 1 to 20 s. The sizes of the resolved macrorestriction fragments were predicted according to an external size standard (Low Range PFG Marker, New England Biolabs Inc.).

### 2.10. Southern Hybridization

PFGE gels were blotted on positively charged membranes using a Vacuum Blotter Model 785 (Bio-Rad). Probes for hybridizations were generated by substituting standard dNTPs with digoxigenin-labelled dNTPs (PCR Dig Probe Synthesis Kit, Applied biosystems, Life Technologies, Burlington, ON, Canada) in the amplification reaction according to the manufacturer’s instructions. Membranes were then probed with digoxigenin-labelled PCR products for the genes *asa1*, *bcrA* and *traB*. Pre-hybridizations and hybridizations were carried out at 71 °C for 30 min and 18 h, respectively, in hybridization buffer with subsequent washings, as recommended by the manufacturer. After post-hybridization washing of membranes, the colorimetric method (NBT/BCIP substrate solution, Applied Biosystems, Life Technologies) was used to detect the presence of digoxigenin-labelled probes. PCR products were used as hybridization controls and control DNA dig-labelled as detection control. DNA extract from *E. faecalis* JH2-2 was used as a negative control.

### 2.11. SDS-PAGE and Western Blotting

Induced and non-induced *E. faecalis* strain 543 cells were collected by centrifugation and extracted using the earlier described lysozyme technique [30]. Briefly, for western blot analysis, these extracts were run on SDS-PAGE gels and transferred to nitrocellulose membranes. These were then incubated overnight at 4 °C in a 1:1,000 dilution in blocking buffer (0.5% Tween 20) of the polyclonal anti-AS_44–560_ antibody. Membranes were washed and incubated with a goat anti-rabbit horseradish peroxidase conjugate (Jackson Immunoresearch Laboratories Inc., West Grove, PA, USA) for 1 h. Proteins extract from *E. faecalis* JH2-2 and pre-immune sera (1:1,000) (GenScript Corporation) were used as negative controls. Detection was performed using tetramethylbenzidine substrate (TMB; Sigma-Aldrich).

### 2.12. Statistical Analysis

The fixed effects linear models were used to determine the statistical significance of differences in transfer rates, number of aggregates per field in clumping assays, and pheromone-responsive conjugation experiments with and without antibodies interference. A *t*-test for unequal variances was used when only two conditions were compared. For multiple comparisons between conditions means in linear models, the sequential Bonferroni adjustment procedure was used to determine comparison-wise alpha levels. Statistical analyses were carried out using SAS v. 9.2 (SAS Institute Inc., Cary, NC, USA). Differences with *p* values of <0.05 were considered significant.

## 3. Results

### 3.1. Conjugal Transfer and Analysis of Transconjugants

**Figure 1:**
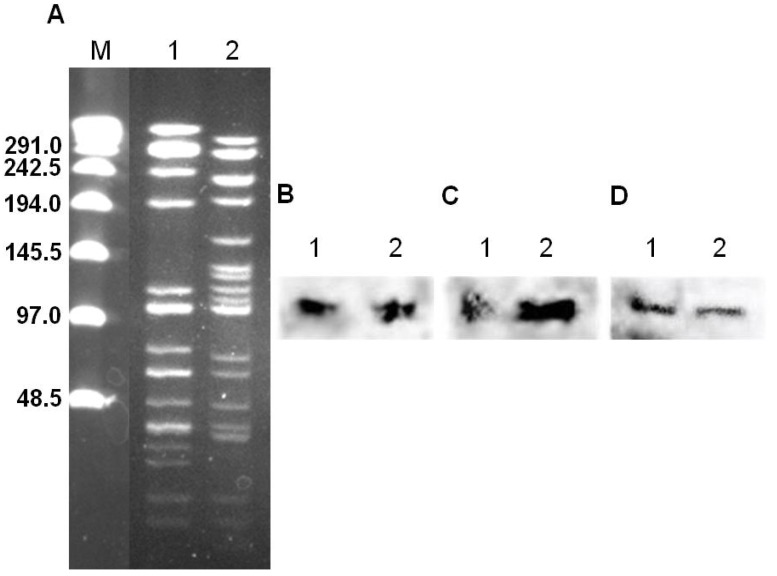
PFGE and hybridization of strains *E. faecalis* 543 and T543-1.

Filter mating assays from donor strain *E. faecalis* 543 to the recipient strain *E. faecalis* JH2-2 revealed conjugative transfer of *asa1*, *bcrRAB* and *traB*. The presence of these genes within the transconjugants was confirmed by PCR, Southern hybridization and sequencing. The *asa1*, *bcrA* and *traB* probes all hybridized on one band of ca. 115 kb in *E. faecalis* strains 543 and T543-1 (Figure 1), demonstrating a colocalization of all genes. No hybridization was observed with the recipient strain *E. faecalis* JH2-2 (data not shown). This is indicative of plasmid colocalization of *asa1* and *bcrRAB* genes in T543-1. BLAST analysis of the sequenced *asa1* gene resulted in identities ranging from 91% to 95% toward 14 different AS genes and 11 of those belong to 10 different pheromone-responsive plasmids and one pathogenicity island (accession number AF454824) in *E. faecalis*. Sequencing of the *traB* gene from *E. faecalis* 543 was also analyzed in BLAST and gave an identity of 98% with the *traB* gene from pJM01 of *E. faecalis* AR01/DGVS [17]. Furthermore, identities of 69% and 70% were observed with three others *traB* genes from pheromone-responsive plasmids pAD1 [31], pTEF1 [32], and pTW9 (Accession number AB563188). The bacitracin resistance genes gave identities of 95% for *bcrR*, 83% for *bcrA* and 87% for *bcrB* with the previously described pJM01 [16]. Regions above *bcrB* and beyond *bcrR* were also examined. Sequence analysis of the region beyond *bcrR* resulted in identities from 94% to 100% to insertion sequence IS*1216*-like transposase gene from pJM01 and pTW9. The region above *bcrB* corresponded in an identity of 82% with the *bcrD* gene from both pTW9 and pJM01. *E. faecalis* strains 543, JH2-2 and T543-1were further characterized by microarray and MIC testing to bacitracin. A MIC to bacitracin of >2,048 µg/mL was obtained for both strains 543 and T543-1. The recipient strain JH2-2 had a MIC of 32 µg/mL. Plasmid stability of the donor strain without selective pressure resulted in plasmid curing after nine broth passages. When transferred to the recipient strain, plasmid of T543-1 was then stable as far as 50 passages in broth. Microarray analysis revealed that bacitracin resistance genes *bcrRAB* and MLS_B_ resistance gene *ermB* (also referred to as *ermAM*) were identified only in the donor strain 543 and one of its transconjugant, T543-1. The tetracycline *tetO* gene was only detected in the donor strain 543. The recipient strain JH2-2 was negative for all these genes. Taxonomic genes *atpA* (alpha unit of ATP synthase)*, ddlfs* (D-Ala: D-Ala ligase) and *pheS* (phenylalanine-tRNA synthetase α chain) and virulence genes *cpd1* (pheromone cPD1 lipoprotein), *cad1* (pheromone cAD1 precursor lipoprotein), *cCF10* (pheromone cCF10 precursor lipoprotein), *agrBfs* (AgrBfs protein of *E. faecalis* involved in gelatinase biosynthesis activating pheromone), *efaAfs* (endocarditis specific antigen of *E. faecalis*) and *gelE* (gelatinase) were also identified by microarray in the donor strain 543, the recipient JH2-2 and T543-1. The sex pheromone gene *cob* (pheromone cOB1 precursor) was only detected in the recipient strain JH2-2 and T543-1. Miroarray data indicates that in addition to *bcrRAB* genes, the *erm*(B) gene was also able to be transferred from the donor to the recipient strain.

PFGE (A) and Southern blot with Dig-labelled probes *traB* (B), *asa1* (C), and *bcrA* (D). Wells loaded with the donor strains *E. faecalis* 543 (1) and the *E. faecalis* T543-1 (2); molecular weight marker (M) (in kb).

### 3.2. Antisera Titer and Specificity

**Figure 2:**
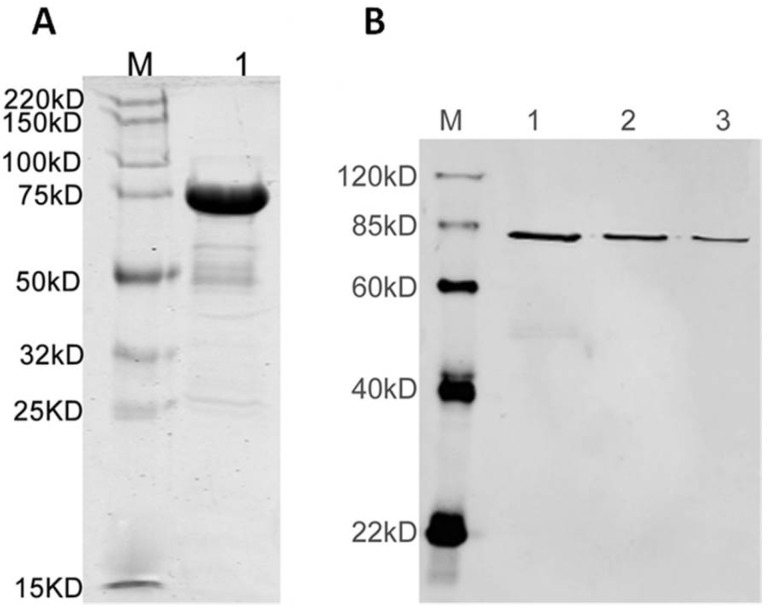
SDS-PAGE and western blot of the purified protein AS.

**Figure 3:**
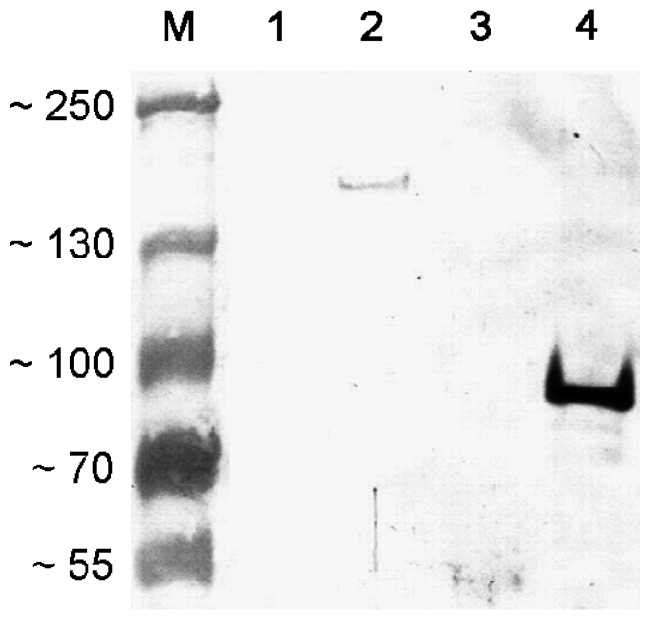
Western blot on *E. faecalis* protein extracts and purified Agg protein.

Antisera titre after the third immunization was 1:152,000 with a S/N (sample/blank) ≥ 2.1. Protein purity was estimated at 85% with a Coomassie blue-stained SDS-PAGE gel and specificity of the polyclonal anti-AS_44–560_ on the purified protein was confirmed by western blot (Figure 2). Detection of the expressed AS protein with the polyclonal anti-AS_44–560_ was also demonstrated using pheromone induced *E. faecalis* strain 543 (Figure 3). No reaction with cell surface proteins was observed with the pre-immune sera (Figure 3).

(**A**) SDS-PAGE: Line M, molecular marker; Line 1, 5 µg of purified protein AS. (**B**) Western blot: Line M, molecular marker; Line 1, 100 ng protein; Line 2, 50 ng protein; Line 3, 10 ng protein. Primary antibodies: rabbit antiserum after 4th immunisation 1:1000. Secondary antibodies: IRDye TM800 conjugated goat anti-rabbit IgG 1:7500.

Hybridization with antiserum Agg 1:1000. Wells loaded with: (1) *E. faecalis* JH2-2 protein extract; (2) *E. faecalis* 543 induced protein extract; (3) *E. faecalis* 543 uninduced protein extract; (4) ca. 10 ng of purified Agg protein; (M) PageRuler prestained protein ladder in kDa.

### 3.3. Interference in the Formation of Aggregates Using Anti-AS_44–560_ Antibodies

**Figure 4:**
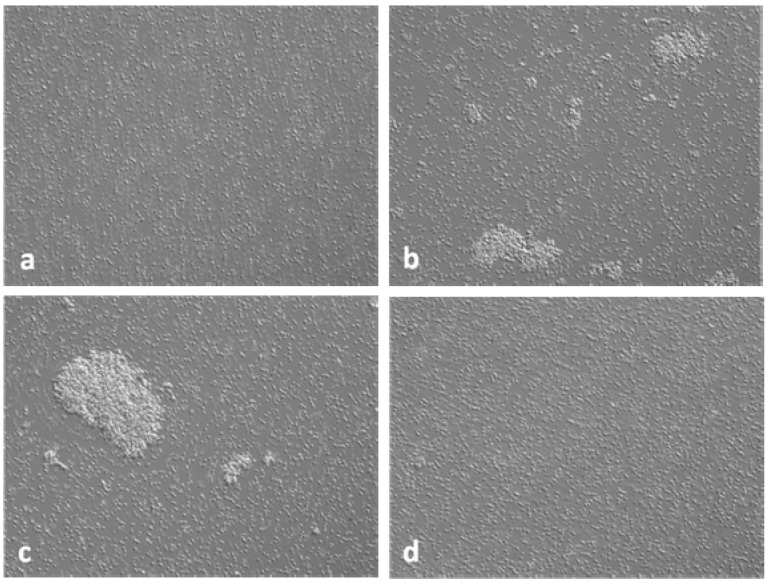
Clumping assays with *E. faecalis* 543 induced and *E. faecalis* JH2-2.

**Figure 5:**
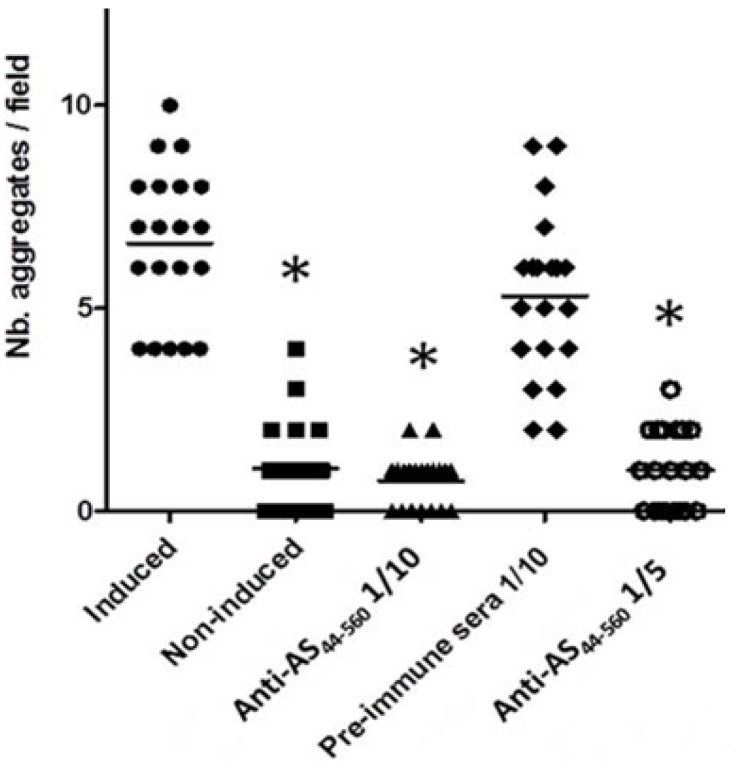
Interference in the formation of aggregates of *E. faecalis* 543 using anti-AS_44–560_ antibodies by microscopy.

The capacity of the pheromone-induced *E. faecalis* strain 543 to aggregate as compared to the non-induced strain was demonstrated in clumping assays (data not shown). When mixed with the recipient strain *E. faecalis* JH2-2, the number of aggregates per field was significantly higher after 20 min compared with the non-induced state (Figure 4(a,b) and Figure 5). A significant reduction in formation of aggregates was observed when the polyclonal anti-AS_44–560_ was added in the pheromone-responsive conjugation experiments as compared to the induced state (positive control) (*p* < 0.0001) (Figure 4(d) and Figure 5). This reduction was still observed after one and two hours of mating assays (data not shown). Aggregation was observed when using the pre-immune serum (Figure 4(c) and Figure 5).

(**a**) Non-induced; (**b**) Induced; (**c**) with pre-immune serum; (**d**) with antiserum Agg 1/10. Magnification of 400×. The same observation was made with antiserum Agg 1/5 as for the dilution 1/10 (data not shown).

Quantification was made on twenty fields for each condition. Straight lines represent the average of aggregates by field. * Significant differences were observed compared to induced state (*p* < 0.05).

### 3.4. Interference in Pheromone-Responsive Conjugation Using Anti-AS_44–560_ Antibodies

Interference of anti-AS_44–560_ antibodies in pheromone-responsive conjugation was demonstrated by a reduction in horizontal transfer of *asa1* and *bcr* genes using *E. faecalis* strain 543 as the donor and *E. faecalis* JH2-2 as the recipient strain (Table 1). The transfer rate to *E. faecalis* JH2-2 from the induced state of *E. faecalis* strain 543 was significantly higher compared to the non-induced (*p* < 0.0001), indicating a pheromone-responsive conjugation process. When anti-AS_44–560_ antibodies (1:10) was added to the above mixtures, the transfer rate was significantly lower as compared to induced-cells without antibodies (*p* < 0.0001). However, transfer rates of uninduced-cells without antibodies (*p* = 0.003) were significantly lower as compared to induced-cells with antibodies (Table 1). Interference of anti-AS_44–560_ antibodies on plasmid transfer was also tested with two other dilutions (1:5 and non-diluted) and with longer mating time (one and two hours), which yield similar results. No significant differences were observed with the addition of pre-immune serum (*p* = 0.19) meaning that the complement or any other molecules of the pre-immune serum were not able to interfere with the pheromone conjugation.

**Table 1:** Interference in the transfer frequencies of plasmid encoding high-level bacitracin resistance of the donor *E. faecalis* strain 543 to the recipient strain *E. faecalis* JH2-2 tested by pheromone-responsive mating experiments with or without addition of antibodies.

Exposure to pheromone ^a^	Antibodies	Transfer frequency in 20-min mating (no. of transconjugants per donor cell) ^c^
+ ^b^	None	4.6 × 10^−3^
-	None	2.2 × 10^−6^
+	pre-immune serum	8.3 × 10^−3^
+	None	4.6 × 10^−3^
+	polyclonal anti-AS_44–560_	4.4 × 10^−5^
+	None	7.5 × 10^−2^
-	None	6.3 × 10^−7^

^a^ +, exposed; -, not exposed; ^b^ Culture filtrate of JH2-2 was used as the pheromone; ^c^ Transfer rates are represented by an average of three independent replicates.

## 4. Discussion

*E. faecalis* is indigenously capable of acquiring, accumulating, and sharing extrachromosomal elements encoding genes that contribute to pathogenesis and antimicrobial resistance [33]. This species has a highly specific and efficient plasmid transfer system called pheromone-responsive which implies the expression of an AS on its surface [11]. The AS binds to a receptor on the surface of the recipient cell to form aggregates and to enhance the conjugation process [15]. This system has also been associated to virulence and antimicrobial resistance [10,12]. In the past years, inhibition of self-clumping of *E. faecalis* expressing AS by an anti-N-terminal AS serum has been demonstrated [34]. The correlation between the ability to aggregate and plasmid transfer efficiency was observed using insertion mutants of the aggregation protein [18]. Moreover, a previous study has used monoclonal anti-Asc10 (AS of pCF10) antibodies and polyclonal F’ab anti-Asc10 to demonstrate the interaction between the AS and the binding substance involved in the pheromone-responsive conjugation of pCF10 and its transfer [15].

The current study further evaluated the *E. faecalis* conjugation pheromone-responsive interference process. It first identified a pheromone-responsive plasmid encoding high-level bacitracin resistance in a previously studied multidrug resistant *E. faecalis* strain 543 [19]. After induction with pheromones produced by the recipient strain *E. faecalis* JH2-2, clumping of the donor *E. faecalis* strain 543 was demonstrated as well as high transfer frequencies in short time broth mating. Conjugative transfer of *asa1*, *traB* and *bcrRAB* genes and their co-localization was also demonstrated in the transconjugant strain T543-1 on a plasmid band of 115 kb. Sequencing of the *asa1* gene encoding for an AS of *E. faecalis* strains 543 and T543-1 further confirmed the association of this genetic element to a pheromone-responsive plasmid. More significantly, this study presents the first evidence that a polyclonal antiserum AS_44–560_ can significantly interfere with the horizontal transfer of a pheromone-responsive plasmid encoding high-level bacitracin resistance.

Sequence analysis of *asa1*, *traB* and *bcrRABD* genes of strain T543-1 indicated a possible pJM01-like pheromone-responsive plasmid. In a previous study, the pJM01 was proposed to be pheromone-responsive based on the presence of negative regulator proteins of the pheromone response, TraA and TraB [17]. This plasmid encodes for the *bcrABD* genes and its regulator, *bcrR*, and the tetracycline resistance gene, *tetM*, and has a molecular weight of 72 kb [16,17]. The pJM01 slightly differs from the one described in this study because their molecular weights are different and the *tetM* gene was not present in any of our tested strains. The variable plasmid content is likely due to rearrangements which occur during the conjugation process [35]. At first, *bcrD* was not detected by PCR on the plasmid. The PCR primers used for the detection of *bcrD* from a previous study [19] amplified successfully part of this gene in the positive *bcr* genes control *E. faecalis* AR01/DGVS [16]. Sequence analysis of the region above *bcrB* revealed an identity of 82% between the *bcrD* gene of this study with the ones of pJM01 and pTW9. The negative PCR amplification with the previously described primers is likely due to the average degree of homology with the *bcrD* of this study. Plasmid stability of the donor strain *E. faecalis* 543, without antimicrobial selective pressure, resulted in plasmid curing after nine passages and over 50 passages in the transconjugant strain. It is reported that plasmid partitioning and addiction systems are responsible for the stability, copy number and the maintenance of plasmids within a bacterial population [36]. A *par* addiction system in *E. faecalis* plasmid pAD1 has been previously described [37]. These results suggest the presence of an addiction module or partition system in the 115-kb plasmid of this study. Also, the sequence of the region beyond *bcrR* which corresponds to an IS*1216*-like transposase gene is likely indicative of transposon-encoded *bcr* genes. Thus, dissemination of high-level bacitracin resistance might occur in *E. faecalis* isolates by horizontal gene transfer involving both transposon and pheromone-responsive conjugation. It has also been described that this type of conjugation could easily play a role in delivering transposons and mobilizing other elements able to replicate in the recipient microorganism [38,39].

Clumping of strain 543 was observed with a light microscope after induction with pheromones produced by *E. faecalis* JH2-2. Clumping was not visible to the naked eyes. This is consistent with previous studies which were not able to observe aggregates by naked eyes [10,40]. Four pheromone genes were detected by microarray in the strain *E. faecalis* JH2-2. The pJM01-related pheromone-responsive plasmid described in this study likely responded to one of those to form aggregates. This non-specific pheromone response likely explains the levels of aggregation by the donor strain *E. faecalis* 543. It has been described that one pheromone could trigger a response to different pheromone-responsive plasmids [34] and that different levels of aggregation were reported for different strains using pheromones from the same producer [10]. In addition, the presence of *gelE*, a gelatinase, in the donor strain *E. faecalis* 543 and the recipient strain *E. faecalis* JH2-2 could also reduce the aggregation process because this enzyme has been shown to lower the pheromone titre in the supernatant and to degrade misfolded proteins such as AS on the cell surface [41,42].

A significant reduction of both aggregation and plasmid transfer was observed after the addition of the polyclonal antiserum AS_44–560_. This is in contrast with a previous study [43] which observed that whole antibodies against an AS, named Asc10, promoted an increased aggregation between Asc10-expressing cells. However, when the Fabs molecules which lack ability to cross-link antigens were used the aggregation between bacterial cells was reduced. Clumping inhibition was also observed with an anti-N-terminal AS serum against an *E. faecalis* isolate containing the sex pheromone plasmid pAD1 [34]. A study also reported a significant reduction of plasmid transfer using a polyclonal anti-AS_44–560_ in mating experiments [15]. Clumping inhibition was proposed to be due to steric hindrance since antibodies and adhesins are in the same size range of about 10 nm and 18 nm, respectively [34]. The antiserum used in this study detected only one protein, extractable from induced, but not from uninduced cells, indicating that the immunological response observed was specific. Furthermore, plasmid transfer reduction correlated well with clumping inhibition. Moreover, a significant reduction was still observed after one and two hours of conjugation indicating good interaction between the antibodies and the expressed AS. Interference in the *E. faecalis* pheromone-responsive conjugation with the aim of reducing horizontal transfer of antimicrobial resistance is of interest because of the clinical importance of antimicrobial resistance in enterococci and their capacity to transfer their resistance genes. Recently, one report has shown that a pheromone responsive plasmid, pLG2 which encodes for MLSb resistances, likely promoted the intra- and interspecies genomic horizontal transfer of an entire pathogenicity island of 153 kb containing several virulence factors including the enterococcal surface protein (esp) from the chromosome of *E. faecalis* strain UW3114 [44]. Thus, interference would then be of major interest to prevent both the acquisition of antibiotic resistance and some pathogenicity traits. Further studies are needed to determine the efficiency of such interference on *E. faecalis* bacitracin resistance transfer in an *in vitro* polymicrobial model such as biofilms. Also, passive immunization experiments using the polyclonal antiserum AS_44–560_ would demonstrate if a significant reduction of plasmid transfer can be observed *in vivo*.

## 5. Conclusions

In summary, the current study provides the evidence of interference in the *E. faecalis* conjugation pheromone-responsive process encoding for high-level bacitracin resistance. Specifically, our results demonstrated conjugative transfer and co-localization of *asa1*, *traB* and *bcrRAB* genes in strain T543-1 on a plasmid band of 115 kb. Our results further indicated a significant reduction of both aggregation and plasmid transfer processes after the addition of the polyclonal antiserum AS_44–560_. The investigation of reducing the horizontal transfer of antibiotic resistant genes in biofilms will be a challenging task for future work.
